# Aortic stiffening and its impact on left atrial volumes and function in patients after successful coarctation repair: a multiparametric cardiovascular magnetic resonance study

**DOI:** 10.1186/s12968-016-0278-6

**Published:** 2016-09-12

**Authors:** Inga Voges, Julian Kees, Michael Jerosch-Herold, Hannes Gottschalk, Jens Trentmann, Christopher Hart, Dominik D. Gabbert, Eileen Pardun, Minh Pham, Ana C. Andrade, Philip Wegner, Ines Kristo, Olav Jansen, Hans-Heiner Kramer, Carsten Rickers

**Affiliations:** 1 Department of Congenital Heart Disease and Paediatric Cardiology, University Hospital of Schleswig-Holstein, Campus Kiel, Arnold-Heller-Str. 3, Haus 9, 24105 Kiel, Germany; 2 Department of Radiology, Brigham & Women’s Hospital and Harvard Medical School, 75 Francis Street, Boston, MA 02115 USA; 3 Department of Diagnostic Radiology, University Hospital of Schleswig-Holstein, Campus Kiel, Arnold-Heller-Straße 3, 24105 Kiel, Germany

**Keywords:** Aortic coarctation, Left ventricular diastolic function, Pulse wave velocity, Aortic distensibility, Arterial stiffness

## Abstract

**Background:**

The increased cardiovascular morbidity of adults with late repair of aortic coarctation (CoA) has been well documented. In contrast, successful CoA repair in early childhood has a generally good prognosis, though adverse vascular and ventricular characteristics may be abnormal, which could increase long-term risk. This study sought to perform a comprehensive analysis of aortic elasticity and left ventricular (LV) function in patients with aortic coarctation (CoA) using cardiovascular magnetic resonance (CMR). In a subgroup of patients, we assessed structure and function of the common carotid arteries to probe for signs of systemic vascular remodeling.

**Methods:**

Fifty-one patients (median age 17.3 years), 13.9 ± 7.5 years after CoA repair, and 54 controls (median age 19.8 years) underwent CMR.

We determined distensibility and pulse wave velocity (PWV) at different aortic locations. In a subgroup, common carotid artery distensibility, PWV, wall thickness and wall area were measured. LV ejection fraction (EF), volumes, and mass were measured from short axis views. Left atrial (LA) volumes and functional parameters (LAEF_Passive_, LAEF_Contractile_, LAEF_Reservoir_) were assessed from axial cine images.

**Results:**

In patients distensibility of the whole thoracic aorta was reduced (*p* < 0.05) while PWV was only significantly higher in the aortic arch (*p* < 0.01). Distensibility of the descending aorta at the level of the pulmonary arteries and PWV in the descending aorta, both correlated negatively with age at CoA repair. LA volume before atrial contraction and minimal LA volume were higher in patients (*p* < 0.05). LAEF_Passive_ and LAEF_Reservoir_ were reduced (*p* < 0.05), and LAEF_Reservoir_ correlated negatively with aortic arch PWV (*p* < 0.05). LVEF, volumes and mass were not different from controls. Carotid wall thickness and PWV were higher in patients compared to controls (*p* < 0.05).

**Conclusions:**

Patients after CoA repair have impaired bioelastic properties of the thoracic aorta with impact on LV diastolic function. Reduced descending aortic elasticity is associated with older age at time of CoA repair. The remodeling of the common carotid artery in our sub-study **s**uggests systemic vessel wall changes.

## Background

Despite successful surgical repair, patients with aortic coarctation (CoA) have a higher cardiovascular morbidity and mortality compared to the healthy population [1, 2]. Early vascular changes have been found [3–5] that may lead to adverse left ventricular (LV) remodeling and even dysfunction. Adult CoA patients have been shown to have poor echocardiographic LV long axis function, which was related to older age at time of intervention and increased aortic stiffness [6]. Cardiovascular magnetic resonance (CMR) has revealed that LV mass was elevated in young CoA patients, which was associated with impaired aortic bioelasticity [7].

CMR has become an important imaging tool in the long-term follow-up of CoA patients because it is well validated for measuring ventricular volumes, mass and parameters describing systolic function, and it has the ability to perform high resolution imaging of the cardiovascular anatomy in 3-dimensional space [8, 9]. Recently, it has been shown that CMR can provide quantitative data on left atrial (LA) volumes which can be used as markers of LV diastolic function [10, 11]. Furthermore, CMR allows accurate assessment of regional vascular distensibility, pulse wave velocity (PWV) [12–14], as well as structural vascular changes, for instance in the carotid arteries [15, 16].

For the current study, we hypothesized that in CoA patients after surgical repair the thoracic aorta shows increased stiffness which impacts the LV function. In a sub-study, we wanted to determine whether CMR can detect systemic changes of vascular bioelasticity and structure, and focused for this on the common carotid artery, the major source of cerebral vascular supply.

## Methods

### Patients

Fifty-one patients after surgical repair were consecutively recruited during follow-up at our institution. Exclusion criteria were evidence of a more than mild re-CoA (mean gradient >20 mmHg), mitral valve stenosis (mean gradient >8 mmHg) and more than mild aortic or mitral valve regurgitation, all assessed by echocardiography.

In patients and controls at least three blood pressure (BP) measurements were taken at the time of CMR. In children blood pressure percentiles were calculated using the fourth report from the National High Blood Pressure Education Program, Working Group on Children and Adolescents from the US National Institutes of Health, USA [17]. In adults (≥18 years) blood pressure was classified using the 2013 guidelines from the task force for the management of arterial hypertension of the European Society of Hypertension and of the European Society of Cardiology [18]:

1. Children (<18 years): Stage 1 hypertension: systolic and/or diastolic BP ranging from 95^th^ to 99^th^ percentile plus 5 mmHg. Stage 2 hypertension: systolic and/or diastolic BP >99^th^ percentile plus 5 mmHg.
2. Adults (≥18 years): Stage 1 hypertension: systolic BP ranging from 140 to 159 mmHg and/or diastolic BP ranging from 90 to 99 mmHg. Stage 2 hypertension: systolic BP ranging from 160 to 179 mmHg and/or diastolic BP ranging from 100 to 109 mmHg.

For comparison to patients, heart-healthy, age-matched controls were recruited among outpatients, medical students, and hospital staff.

In CoA patients <8 years, sedation with propofol and midazolame was used for the CMR study. Monitoring of electrocardiogram, BP and oxygen saturation using a CMR compatible monitoring system (Precess™, Invivo, Florida, USA) was performed in patients and controls.

Written informed consent was obtained from all patients, controls, parents or guardians, as appropriate. The study protocol was approved by the ethics committee of the medical faculty of the Christian Albrechts University in Kiel (Nr. A104/10).

### CMR acquisition

CMR studies were done with a 3.0-Tesla scanner (Achieva 3.0 T, Philips Medical Systems, Netherlands) with a phased-array coil (SENSE™ Cardiac coil, Philips Medical Systems, Netherlands). In a subgroup of 11 patients and 13 controls, common carotid artery imaging was performed using an 18-element head and neck coil (SENSE Neurovascular coil 18 elements, Philips Medical Systems, Netherlands). This head and neck coil was available only for the last patients enrolled (*n* = 11).

Gradient echo cine CMR with retrospective gating was applied to measure aortic cross-sectional areas (CSA), to describe dimensions at end-systole and end-diastole, and to analyse aortic distensibility. The angulation of the images slices was adjusted to locally intersect the axis of the aorta at, or close to (~ ±10°) a right angle (as we used stacks of parallel slices for sections of the ascending and descending aorta to speed up scan planning and acquisition, it was not possible to intersect the aorta in all slices at exactly a right angle). The scan parameters were as follows: Field of view (FOV) 280 × 224 mm, matrix size 149 × 116, voxel size 1.88 × 1.94 × 6 mm, TR/TE = 4.4/2.5 ms, 25 cardiac phases, number of repetitions: 2, scan duration per slice: 15 s. To assess LA volumes and functional parameters indicating LV diastolic function we applied axial gradient-echo cine sequences with retrospective ECG gating [10, 11]. The sequence parameters were as follows: FOV 280 × 224 mm, matrix size 149 × 116, voxel size 1.88 × 1.94 × 6 mm, TR/TE = 4.4/2.5 ms, 25 cardiac phases, number of repetitions: 2, scan duration per slice: 15 s.

To evaluate aortic PWV we performed two-dimensional phase-contrast (PC) imaging with through-plane velocity encoding (VENC = 2 m/s) and retrospective ECG gating for three locations in the ascending (AAo) and descending aorta (DAo; parameters: FOV 270 × 270 mm, matrix size 165 × 193, voxel size 1.64 × 1.4 × 7 mm, TR/TE = 4.4/2.7 ms, velocity encoding strength 200 cm/s, 80 phases, k-space segmentation factor of 1, SENSE factor 1.8, scan duration 45–75 s). The maximal temporal resolution corresponded to 2 × repetition time, equaling 9 ms. The first image plane allowed simultaneous flow measurement in the AAo and the proximal DAo using a slice plane intersecting the aorta at both locations at an approximately right angle. The second image plane was perpendicular to the DAo at the level of the diaphragm.

LV volumes and systolic function were measured using a gradient-echo cine sequence in the short-axis plane. Imaging parameters included the following: FOV 330 × 330 mm, matrix size 176 × 190, voxel size 1.88 × 1.74 × 6 mm, TR/TE = 3.7/1.8 ms, 25 cardiac phases, number of repetitions: 2, scan duration: 3–6 min.

Gradient-echo cine imaging of the neck with retrospective ECG gating was used to measure carotid CSA areas as a basis for calculating distensibility. The parameters were: FOV 280 × 224 mm, matrix size 149 × 116, voxel size 1.88 × 1.94 × 6 mm, TR/TE = 4.4/2.5 ms, 25 cardiac phases, number of repetitions 2, scan duration 3–6 min.

For the estimation of carotid artery PWV, flow was measured in the proximal and distal (just below the bifurcation) carotid artery by using a retrospectively-gated PC sequence with the following parameters: FOV 270 × 270 mm, matrix size 165 × 193,voxel size 1.64 × 1.4 × 7 mm, TR/TE = 4.4/2.7 ms, velocity encoding strength 200 cm/s, 80 phases, k-space segmentation factor of 1, SENSE factor 1.8, scan duration 45–75 s; maximal temporal resolution corresponded to 2 × repetition time, equaling 9 ms.

Carotid arterial wall thickness and wall area were assessed by using a multislice T2 dark-blood fast spinecho sequence (parameters: FOV 160 × 160 mm, matrix size 251 × 384, voxel size 0.2 mm, slice thickness 2 mm, TR/TE = 3000/80 ms).

### CMR data analysis

Image analysis was performed with software for cardiac analysis (Extended MR WorkSpace 2.6.3.2 HF3, Philips Medical Systems, Netherlands).

**Fig. 1:**
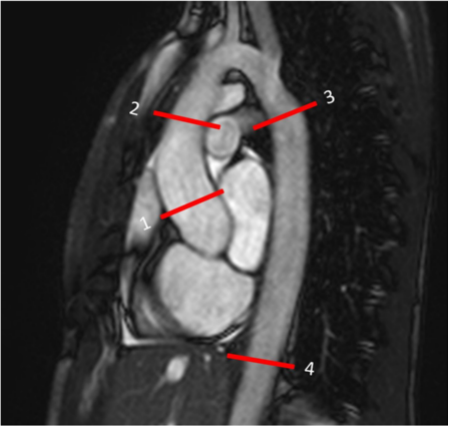
Gradient-echo cine images show assessment of aortic CSA. CSA were measured at four levels: 1) aortic root, 2) AAo, 3) DAo at the pulmonary bifurcation and 4) DAo at the diaphragm

Aortic CSA’s were measured from oblique or double oblique cine im﻿﻿﻿ages at four locations of the thoracic aorta at the time of the minimal and maximal distension of the cardiac cycle. The measuring points were the aortic root, the AAo, the DAo at the level of the pulmonary arteries (aortic isthmus) and the DAo at the diaphragm (Fig. 1). Carotid CSA were measured at the proximal and distal carotid artery. CSA were used to describe aortic anatomy and dimensions as well as to evaluate aortic and carotid distensibility.

Distensibility was calculated according to the following formula [19]:

$$ \left({\mathrm{A}}_{\max}\hbox{-} {\mathrm{A}}_{\min}\right)/\left({\mathrm{A}}_{\min }*\left({\mathrm{P}}_{\min}\hbox{-} {\mathrm{P}}_{\max}\right)\right) $$

where A_min_ is the minimal and A_max_ is the maximal CSA. P_min_ and P_max_ are the systolic and diastolic BP. BP were recorded with a sphygmomanometer during CMR. The cuff was placed around the right upper arm.

**Fig. 2:**
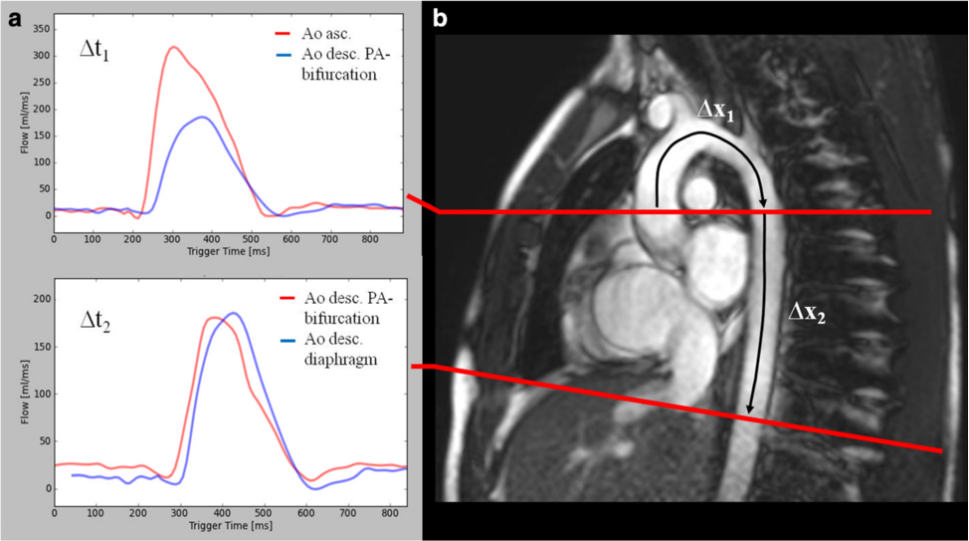
**a** Aortic flow-curves from PC cine imaging. We used the validated method cross-correlation to determine the time delay (∆t) between the proximal and distal flow curves [20]. **b** Sagittal angulated cine image which shows the orientation of scan planes for PC imaging. The distance between the particular positions (∆t) was measured by drawing a curved-line following the midline course of the thoracic aorta

PWV was assessed from PC measurements in two predefined aortic segments. The first segment extended from the AAo to the DAo at the level of the pulmonary arteries, the second segment corresponded to a section of the DAo from the level of the pulmonary arteries to the diaphragm. Furthermore, we measured common carotid artery PWV. Flow versus time curves from PC cine images were obtained and the time delay (Δt, Fig. 2a) of the distal flow curve relative to the proximal flow curve was determined by a validated method, which is based on the cross-correlation between the systolic up-stroke portions of two flow waveforms [20]. The midline distance between the particular positions was measured on angulated sagittal images (Δx, Fig. 2b) and for the carotid artery on images from the time-of-flight angiography. PWV was then calculated by the means of the following equation:

$$ \mathrm{P}\mathrm{W}\mathrm{V}\left(\mathrm{m}/\mathrm{s}\right)=\Delta \mathrm{x}/\Delta \mathrm{t}. $$

LV end-diastolic volume (LVEDV), LV end-systolic volume (LVESV) and LV mass were obtained from short axis cine images by drawing endocardial and epicardial contours at end diastole and end systole. LV stroke volume (SV) was calculated as the difference of LVEDV from the LVESV, and LV ejection fraction (LVEF) was obtained by dividing the LVSV by the LVEDV.

**Fig. 3:**
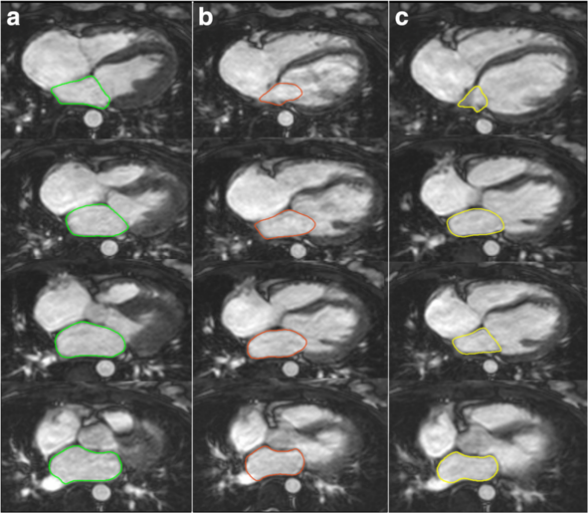
Volumetric assessment of LA volumes on axial cine images: **a** LA-Vol_max_ (*green*). **b** LA-Vol_ac_ (*red*) and **c** LA-Vol_min_ (*yellow*)

LA volumes were quantified using Simpson’s rule and manual planimetry of axial cine images [15]. Tracings were performed at different times in the cardiac cycle (Fig. 3): 1) maximal LA volume before mitral valve opening (LA-Vol_max_), 2) before LA contraction (LA-Vol_ac_) and 3) minimal LA volume at mitral valve closure (LA-Vol_min_). We used this dimensions to measure additional LA volumes and functional parameters [11, 21]:

- LA total emptying volume = LA-Vol_max_ – LA-Vol_min_
- LA passive emptying volume (V_Passive_) = LA-Vol_max_ – LA-Vol_ac_
- LA contractile volume (V_Contractile_) = LA-Vol_ac_ – LA-Vol_min_
- LA passive emptying function (LAEF_Passive_) = (LA-Vol_max_ – LA-Vol_ac_)*100 %/LA-Vol_max_,
- LA contractile emptying function (LAEF_Contractile_) = (LA-Vol_ac_ – LA-Vol_min_)*100 %/LA-Vol_ac_,
- LA reservoir emptying function (LAEF_Reservoir_) = (LA-Vol_max_ – LA-Vol_min_)*100 %/LA-Vol_max_.
- LV and LA volumes were indexed to body surface area (BSA).

**Fig. 4:**
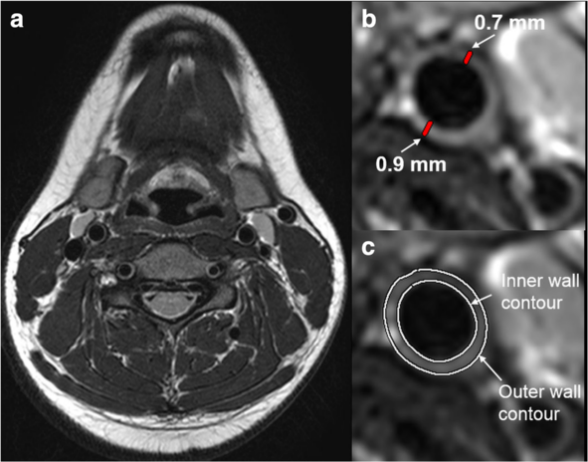
Measurement of carotid wall thickness and area from (**a**) T2 dark-blood fast spinecho images. Wall thickness (**b**) was measured at two positions for each vessel. Wall area (**c**) was determined by drawing an outer and inner contour to measure first the entire vessel area (including the vessel wall) and the lumen area. The lumen area was then subtracted from the vessel area [15]

Carotid wall thickness was measured at two positions and two sites for each carotid artery, respectively [16]. Wall area was assessed by manual tracing of the inner and outer contour of the carotid wall on the T2 dark-blood fast spinecho images as reported previously [15] and shown in Fig. 4.

### Statistical analysis

Statistical analysis was performed using MedCalc Version 12.3.0.0 (MedCalc statistical software, Mariakerke, Belgium) and R (R Foundation for Statistical Computing, Vienna, Austria; URL: http://www.R-project.org/).

To determine if a variable was normally distributed we visually assessed a quantile-quantile plot of the data. All variables except age appeared on the Q-Q plots to be normally distributed, with deviations from the ideal distribution at the tails that were relatively small. All continuous variables were expressed either as mean ± standard deviation if they appeared to be normally distributed, or otherwise as median with range. Differences between patients and controls were compared using the Mann-Whitney-U test. Correlations between variables were measured by Spearman’s rank method. All tests were two-tailed. *P* values of less than 0.05 were considered to indicate statistical significance. Linear regression analysis for distensibility at four aortic locations was performed with linear mixed-effects (LME) methods to account for any intra-patient correlation of distensibility measurements. The model for distensibility included a random intercept, age at the time of repair, age at time of MRI, and measurement location. For the measurement location with 4 levels (root, ascending aorta, isthmus, and descending aorta) we used a so-called “treatment” contrast matrix, where the descending aorta, which was least affected by CoA, served as reference level. LME analysis was performed with the “lme4” package in R(version 1.1-12; URL: http://cran.r-project.org).

## Results

51 patients (median 17.3 years; 0.9–42.3 years) with CoA (median age at repair 1.0 years; 0.01–28.1 years) were recruited for the study. 27 patients were younger than 18 years and 24 patients were 18 years or older. Of the 27 patients, only 6 were younger than 10 years. 26 patients underwent surgery before the age of 1 year (median 0.04 years; 0.01–0.89 years) and 25 patients were older than 1 year at surgery (median 6.5 years; 1.2–28.1 years). 54 individuals served as healthy controls. A previous study from our group in healthy volunteers showed relatively small changes for aortic PWV between the ages of 2 and 28 years [22]. For this reason, we enrolled patients with ages covering a relatively broad range to maximize the chances of detecting an association of PWV and other bioelastic properties with the age at CMR, and distinguish this from any association with age at the time of CoA repair.

Six patients needed interventional (balloon dilatation *n* = 5, stent implantation *n* = 1) treatment of re-CoA at a median time difference of 0.4 (0.5–14) years after surgery. Two patients underwent reoperation, one of them after unsuccessful balloon angioplasty. Sixteen patients had a bicuspid aortic valve without significant stenosis or insufficiency. In seven patients a ventricular septal defect was closed surgically. In addition, there were 3 patients with a small ventricular septal defect and one with a partial anomalous pulmonary venous connection. None of the patients had evidence for re-CoA or an aortic aneurysm shown by CMR during the study.

34 patients had normal BP, 4 patients had stage 1 hypertension and 13 patients needed antihypertensive treatment which was effective at the time of the study. Mean and diastolic BP were not different between patients and controls.

Characteristics of patients and controls are summarized in Table 1.

**Table 1:** Clinical characteristics of CoA patients and controls

Characteristic	Patients (*n* = 51)	Controls (*n* = 54)	*P* value
Age (y)	17.3 (0.9–42.3)	19.8 (2.3–40.1)	0.54
Age at initial surgery (y)	4.2 ± 6.1	NA	NA
Female/Male (n)	18/33	31/23	NA
Weight (kg)	61.8 ± 26.6	58.0 ± 21.3	0.35
Height (cm)	163.7 ± 24.2	165.5 ± 20.9	0.98
BSA (m^2^)	1.7 ± 0.5	1.6 ± 0.5	0.36
Systolic blood pressure (mmHg) ^a^	111.9 ± 15.4	107.0 ± 8.7	0.047
Diastolic blood pressure (mmHg) ^a^	60.5 ± 9.6	61.6 ± 10.5	0.79
Mean blood pressure (mmHg) ^a^	80.1 ± 10.7	79.1 ± 9.8	0.35
Pulse Pressure (mmHg) ^a^	51.5 ± 14.3	45.3 ± 9.0	0.04
Heart rate (bpm) ^a^	74.1 ± 15.7	70.3 ± 16.9	0.38
Medications (n)			
Beta-blockers	7	–	–
ACE inhibitors	6	–	–
Diuretics	3	–	–

Data are presented as mean ± SD or median and range. *P*-Values are from the Mann-Whitney-U test

*ACE* angiotensin-converting enzyme, *BSA* body surface area

^a^ Data were measured at the time of the CMR study

### Regional aortic dimensions and bioelasticity

There were no significant differences in aortic CSA between patients and controls. Patients with a bicuspid aortic valve had an enlarged CSA of the AAo compared to age-matched patients without a bicuspid aortic valve (439.1 ± 101.1 vs. 332.5 ± 88.5 mm^2^/m^2^, *p* = 0.007). The presence of a bicuspid aortic valve had no effect on aortic distensibility or PWV.

**Fig. 5:**
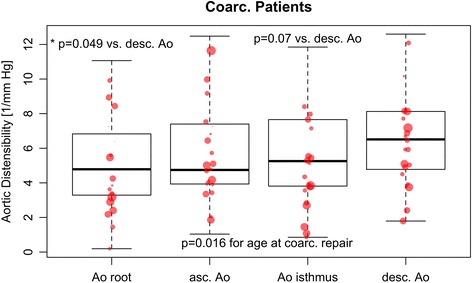
In patients with repaired CoA the distensibility of the aorta was significantly lower in the aortic root and trended lower in the aortic isthmus, compared to the descending aorta. Both age at time of repair and aortic location were included in a linear mixed effects model, and the *p*-values shown in the graph were obtained from this model, which accounts for repeated measurements (at 4 aortic locations) in each patient. Across all locations, the distensibility was lower if the repair was performed at a later age (*p* = 0.016). The size of the data points is proportional to the age at the time of surgery, and larger data points are seen for lower distensibility values, illustrating the significant effect of higher age at time of repair. The DAo was chosen as reference level, as the values there were closest to those observed in normal volunteers

In patients distensibility was significantly lower than in controls at all positions of the thoracic aorta (Table 2). In CoA patients aortic root distensibility was lowest (*p* = 0.05) and trended lower in the aortic isthmus (*p* = 0.07), compared to the descending aorta (Fig. 5). Furthermore, distensibility across the different locations was lower if the repair was performed at a later age (*p* = 0.016; Fig. 5). As patients who had aortic repair later, also tended to be older, both age at time of repair and age at time of CMR were used as predictors in the model for distensibility. Age at the time of MRI did not have a significant effect.

**Table 2:** Comparison of CMR measurements in patients and controls

Variable	Patients (*n* = 51)	Controls (*n* = 54)	*p* Value
Maximal aortic area (mm^2^/m^2^)			
Aortic root	449.5 ± 126.1	440.5 ± 93.0	0.95
Ascending aorta	353.2 ± 104.5	356.3 ± 68.4	0.41
Descending aorta at the isthmus	163.2 ± 61.5	176.9 ± 32.0	0.02
Descending aorta at the level of the diaphragm	151.9 ± 35.3	155.2 ± 38.0	0.82
LVEDV (ml/m^2^)	81.0 ± 15.3	80.1 ± 12.0	0.91
LVESV (ml/m^2^)	30.4 ± 12.0	31.0 ± 6.2	0.28
LVSV (ml/m^2^)	50.6 ± 8.2	49.0 ± 7.9	0.35
LVEF (%)	63.3 ± 8.0	61.3 ± 4.4	0.04
LV mass (g/m^2^)	60.7 ± 14.1	57.5 ± 14.2	0.25
LA Vol_max_ (ml/m^2^)	47.5 ± 10.2	43.2 ± 8.7	0.07
LA Vol_ac_ (ml/m^2^)	32.3 ± 8.0	27.4 ± 5.9	<0.01
LA Vol_min_ (ml/m^2^)	24.6 ± 6.1	20.9 ± 5.1	<0.01
LA Total emptying volume (ml/m^2^)	23.0 ± 6.1	22.5 ± 5.7	0.98
V_Passive_ (ml/m^2^)	15.3 ± 5.0	15.6 ± 4.9	0.50
V_Contractile_ (ml/m^2^)	7.7 ± 3.3	6.5 ± 2.7	0.06
LAEF_Contractile_ (%)	23.7 ± 7.2	23.7 ± 8.1	1.00
LAEF_Passive_ (%)	32.2 ± 8.1	36.9 ± 6.6	<0.01
LAEF_Reservoir_ (%)	48.4 ± 6.9	51.9 ± 6.8	<0.05
Distensibility (10^−3^ mmHg^−1^)			
Aortic root	5.6 ± 3.8	7.4 ± 3.0	<0.01
Ascending aorta	5.8 ± 3.1	8.1 ± 3.6	<0.01
Descending aorta at the isthmus	5.7 ± 3.0	6.8 ± 2.3	<0.01
Descending aorta at the level of the diaphragm	6.8 ± 2.8	8.0 ± 2.8	<0.05
PWV aortic arch (m/s)	4.6 ± 1.7	3.5 ± 0.8	<0.01
PWV descending aorta (m/s)	4.3 ± 1.6	3.9 ± 0.8	0.70

Data are presented as mean ± SD. *P*-Values are from the Mann-Whitney-U test

*LVEF* left ventricular ejection fraction, *LVSV* left ventricular stroke volume, *LVEDV* left ventricular end-diastolic volume, *LVESV* left ventricular end-systolic volume, *LA Vol*_*max*_ maximal left atrial volume, *LA Vol*_*min*_ minimal left atrial volume, *LA Vol*_*ac*_ left atrial volume just before atrial contraction, *LAEF*_*Contractile*_ left atrial contractiale emptying function, *LAEF*_*Passive*_ left atrial passive emptying function, *LAEF*_*Reservoir*_ left atrial reservoir emptying function, *PWV* pulse wave velocity, *V*_*Contractile*_ left atrial contractile volume, *V*_*Passive*_ left atrial passive emptying volume

Aortic arch PWV was significantly elevated in patients compared to controls, whereas PWV in the DAo was not significantly different between patients and controls (Table 2), and this difference remained significant with simultaneous adjustment by age. In controls PWV in the DAo trended higher compared to the aortic arch (+0.45 m/s; *p* = 0.07), but in patients, PWV in the DAo was significantly lower than in the aortic arch (−0.82 m/s; *P* = 0.03), with both comparisons being made with simultaneous adjustment by age (*p* < 1e-5). PWV in the DAo correlated with age at repair (*r* = 0.33, *p* < 0.05), consistent with the effect of age at time of repair that was observed for aortic distensibility.

### LV systolic and diastolic function

LV volumes and mass indexed by BSA were similar in patients and controls (Table 2). LVEF was higher in patients. There was no correlation between reduced aortic distensibility or increased PWV and these LV parameters (*p > 0.05*).

**Fig. 6:**
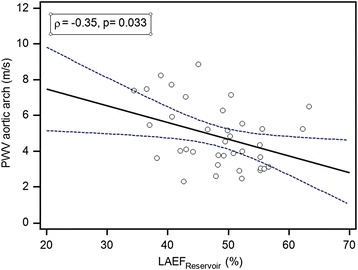
Relation between LAEF_Reservoir_ and aortic arch PWV. The dotted lines represent the borders of the 95 % confidence intervals

LA-Vol_ac_ and LA-Vol_min_ were significantly higher and LV-Vol_max_ trended higher in patients (Table 2). LA-Vol_ac_ correlated with higher aortic arch PWV (*r* = 0.33, *p* < 0.05). Furthermore, LAEF_Passive_ and LAEF_Reservoir_ were significantly reduced in the patient group compared to the control group (Table 2) and LAEF_Reservoir_ correlated negatively with aortic arch PWV (*r* = −0.35, *p* < 0.05, Fig. 6). In patients who underwent surgery after the age of 1 year LAEF_Passive_ was significantly lower compared to patients who were younger than 1 year (29.2 ± 8.9 vs. 34.7 ± 6.5, *p* < 0.05). LAEF_Passive_ was reduced in patients with arterial hypertension compared to patients without arterial hypertension.

### Bioelasticity, wall thickness and wall area of the common carotid artery

Table 3 demonstrates the comparison of carotid artery measurements between patients and controls. PWV, wall thickness and wall area were significantly higher in patients than in controls. Carotid distensibility was not statistically different between both groups.

**Table 3:** Comparison of carotid artery MRI measurements in patients and controls

Variable	Patients (*n* = 11)	Controls (*n* = 13)	*p* Value
Distensibility (10^−3^ mmHg^−1^)			
Proximal right carotid artery	10.4 ± 7.0	5.9 ± 3.8	0.048
Distal right carotid artery	8.3 ± 2.9	7.9 ± 3.0	0.64
Proximal left carotid artery	8.7 ± 5.8	8.1 ± 5.5	0.36
Distal left carotid artery	7.1 ± 4.8	6.2 ± 2.2	0.74
PWV right carotid artery (m/s)	6.8 ± 4.1	3.3 ± 1.5	<0.01
PWV left carotid artery (m/s)	6.9 ± 4.0	4.2 ± 1.7	0.10
Wall area right carotid artery (mm^2^)	19.4 ± 2.4	15.3 ± 2.6	<0.01
Wall area left carotid artery (mm^2^)	19.7 ± 4.1	15.8 ± 1.9	<0.05
Wall thickness right carotid artery (mm)	0.90 ± 0.12	0.75 ± 0.09	<0.01
Wall thickness left carotid artery (mm)	0.90 ± 0.12	0.76 ± 0.08	<0.05

Data are presented as mean ± SD. *P*-Values are from the Mann-Whitney-U test

## Discussion

After successful CoA repair, patients with normal LV systolic function and size, had significantly decreased distensibility of the entire thoracic aorta, and PWV was significantly higher in the aortic arch indicating adverse aortic remodeling. LA markers of left-sided diastolic dysfunction were impaired and correlated partly with the aortic bioelastic markers. Distensibility of the entire aorta was lower if CoA repair was performed at later age.

### Regional aortic bioelasticity

In our patients distensibility was reduced in all parts of the thoracic aorta compared to controls. Consistent with this, PWV was increased in the aortic arch, but not in the DAo.

Ou et al. evaluated aortic bioelasticity in 40 normotensive CoA patients by CMR and found a reduced distensibility of the AAo but not of the DAo at the pulmonary bifurcation [7]. Two other echocardiographic studies showed an impaired AAo distensibility and increased stiffness index before CoA repair and also after successful operation but elastic properties of the abdominal DAo were not different from controls [3, 4]. The discrepancies might be explained by difference in median patient age as our cohort was 6–18 years older compared to the cited studies. Additionally, Ou et al. excluded patients with arterial hypertension or on cardiac medication [7]. The mean BP of our patients was not different compared to our controls.

There are several reasons for a reduced aortic bioelasticity in CoA. Histopathological studies by Niwa et al. showed abnormalities of the aortic media, both proximally and distal to the coarctation [23]. The decreased elasticity of the DAo at the pulmonary bifurcation, representing the isthmic area, can additionally be caused by scar formation due to the surgical procedure. In addition, arterial hypertension leads to structural and functional arterial wall alterations [24]. In our cohort we found no differences between patients with (4 pts had stage 1 hypertension, 13 pts under effective antihypertensive treatment) and patients without arterial hypertension which supports the notion that our patients did not have severe arterial hypertension.

Cohen et al., showed more than 25 years ago, that older age at the time of repair contributes to the risk of hypertension [2]. We found a correlation between aortic distensibility at the pulmonary bifurcation as well as PWV in the DAo and age at surgical CoA repair, respectively. The association between age at repair and DAo distensibility remained significant with simultaneous adjustment by age at time of CMR.

### LV systolic and diastolic function

This is the first CMR study which evaluated the relation between aortic bioelasticity and LV systolic and diastolic function in CoA patients. LA size has been shown to be a reliable and important indicator of diastolic dysfunction and it provides prognostic information in various kinds of cardiac diseases [25–27]. In this study, we have not only used maximum LA volume but have measured also several other LA volumes and LA functional parameters to describe LA function during the cardiac cycle and to assess the relative contribution of LA function to LV filling which is dependent on LV diastolic function [26, 27].

We found that LA volumes, LA-Vol_ac_ and LA-Vol_min_, were significantly higher and that LAEF_Passive_ and LAEF_Reservoir,_ all markers of LV diastolic function, were reduced in patients compared to controls. Increased aortic arch PWV correlated with LA-Vol_ac_ and LAEF_Reservoir_. Myocardial mass was similar in patients and controls, which might be explained by the fact that all but 4 patients had normal BP.

Our findings show, that CoA-patients have an impaired LV diastolic function which might result from the impaired aortic bioelastic function, which can increase LV afterload. It has been speculated that an impaired aortic bioelasticity can impair diastolic function through early reflection of the pulse wave leading to increased LV afterload and decreased coronary perfusion [28]. The increased afterload and the decreased coronary perfusion, may compromise myocardial relaxation and promote subendocardial ischemia as well as interstitial fibrosis leading to reduced LV compliance [28–30]. An echocardiographic study of CoA patients by Lombardi et al. recently also demonstrated that an elevated aortic stiffness is linked to diastolic impairment [31]. Using tissue Doppler imaging, Florianczyk et al. also found abnormal LV diastolic mechanics in patients after successful CoA repair, but did not evaluate the aortic bioelasticity [32].

Arterial hypertension is known to cause LV diastolic dysfunction in the longterm [32]. In our patients we found markers for LV diastolic dysfunction despite all but 4 having normal BP. We suppose that already early stages of increased aortic stiffness promotes LV diastolic dysfunction in CoA patients. This may have clinical implications as to avoid cardiovascular risk factors aggravating aortic stiffness.

Patients with diastolic dysfunction have an abnormal relaxation and an increased LV chamber stiffness impairing LV filling [33]. In patients with preserved LV systolic function, diastolic dysfunction is related to poor outcome [34]. The early detection of impaired aortic bioelasticity and diastolic dysfunction may therefore be important for optimal patient management.

### Common carotid artery bioelasticity, wall thickness and wall area

Patients in our substudy showed an increased carotid wall thickness and area as well as a higher PWV while the distensibility as a parameter of wall stiffness was not increased. These findings do not contradict one another. Rather, they demonstrate that bioelasticity, in terms of the elastic modulus of the carotid arteries was not impaired, and the higher PWV in patients can be attributed to the increased carotid wall thickness [35, 36]. For comparisons of PWV’s the effect of vessel wall thickness needs to be taken into account [36].

Two former ultrasound studies found a decreased carotid distensibility after CoA repair [37, 38]. An increased intima-media thickness of the carotid artery, indicating adverse vascular remodeling, has been reported by ultrasound studies [39]. Our results of increased wall thickness and area in patients are in line with these findings. They underline, that the increased arterial stiffness is not restricted to the aorta. The alterations found in the carotid arteries may be rather representative for other parts of the systemic vascular bed.

### Study limitations

The present study has several limitations. Although our study included 51 patients and 54 controls, only in a subgroup of 11 patients and 13 controls the carotid artery was examined. However, as significantly different results were obtained even in these small groups, carotid wall changes must be considered as severe. As the range of BP was rather small with only a few hypertensive patients (*n* = 4) we could not analyze the effect of BP on anatomical and functional vascular changes.

## Conclusions

Patients after CoA repair show reduced aortic bioelasticity of the entire thoracic aorta which was associated with older age at repair. LV diastolic function was impaired, despite normal BP in most patients, which suggests that the increase of aortic stiffness sufficiently increases the LV afterload to induce LV diastolic dysfunction. Monitoring of aortic bioelastic and LV functional parameters is therefore important during follow-up. In addition, our study supports the strategy to treat CoA patients expeditiously after initial diagnosis.

## Abbreviations

AAo: Ascending aorta
BP: Blood pressure/pressures
CMR: Cardiovascular magnetic resonance
CoA: Aortic coarctation
CSA: Cross-sectional area/areas
DAo: Descending aorta
FOV: Field of view
LA: Left atrium/atrial
LAEF_Contractile_: Left atrial contractile emptying function
LAEF_Passive_: Left atrial passive emptying function
LAEF_Reservoir_: Left atrial reservoir emptying function
LA-Vol_ac_: Left atrial volume before left atrial contraction
LA-Vol_max_: Maximal left atrial volume
LA-Vol_min_: Minimal left atrial volume
LV: Left ventricle/ventricular
LVEDV: Left ventricular enddiastolic volume
LVEF: Left ventricular ejection fraction
LVESV: Left ventricular endsystolic volume
LVSV: Left ventricular stroke volume
PC: Phase contrast
PWV: Pulse wave velocity
VENC: Velocity encoding
V_passive_: Left atrial passive emptying volume
V_contractile_: Left atrial contractile volume
